# Estradiol and Estrogen Receptor Agonists Oppose Oncogenic Actions of Leptin in HepG2 Cells

**DOI:** 10.1371/journal.pone.0151455

**Published:** 2016-03-16

**Authors:** Minqian Shen, Haifei Shi

**Affiliations:** Department of Biology, Cell, Molecular, and Structural Biology, Miami University, Oxford, Ohio, United States of America; II Università di Napoli, ITALY

## Abstract

Obesity is a significant risk factor for certain cancers, including hepatocellular carcinoma (HCC). Leptin, a hormone secreted by white adipose tissue, precipitates HCC development. Epidemiology data show that men have a much higher incidence of HCC than women, suggesting that estrogens and its receptors may inhibit HCC development and progression. Whether estrogens antagonize oncogenic action of leptin is uncertain. To investigate potential inhibitory effects of estrogens on leptin-induced HCC development, HCC cell line HepG2 cells were treated with leptin in combination with 17 β-estradiol (E2), estrogen receptor-α (ER-α) selective agonist PPT, ER-β selective agonist DPN, or G protein-coupled ER (GPER) selective agonist G-1. Cell number, proliferation, and apoptosis were determined, and leptin- and estrogen-related intracellular signaling pathways were analyzed. HepG2 cells expressed a low level of ER-β mRNA, and leptin treatment increased ER-β expression. E2 suppressed leptin-induced HepG2 cell proliferation and promoted cell apoptosis in a dose-dependent manner. Additionally E2 reversed leptin-induced STAT3 and leptin-suppressed SOCS3, which was mainly achieved by activation of ER-β. E2 also enhanced ERK via activating ER-α and GPER and activated p38/MAPK via activating ER-β. To conclude, E2 and its receptors antagonize the oncogenic actions of leptin in HepG2 cells by inhibiting cell proliferation and stimulating cell apoptosis, which was associated with reversing leptin-induced changes in SOCS3/STAT3 and increasing p38/MAPK by activating ER-β, and increasing ERK by activating ER-α and GPER. Identifying roles of different estrogen receptors would provide comprehensive understanding of estrogenic mechanisms in HCC development and shed light on potential treatment for HCC patients.

## Introduction

Hepatocellular carcinoma (HCC) is the most common primary carcinoma in the liver and the fourth most common cancer worldwide with high malignancy. The incidence and mortality rate of HCC continue to increase in the USA [1]. The common risk factors of developing HCC include obesity, nonalcoholic fatty liver disease, chronic alcohol consumption, viral hepatitis infection, cirrhosis, and aflatoxin exposure. Among the aforementioned risk factors, the rapid increase in obesity has become the prime cause of HCC, outweighing alcohol- or virus-related etiology [2]. Epidemiological and clinical studies indicate that people with a body mass index (BMI) over 35 have greater risk for developing HCC, and obesity can precipitate other risk factors for HCC [3–5].

Leptin is a 16-KD protein primarily secreted by white adipose tissue, and its level increases in obese animals including humans. Leptin is involved in the regulation of many physiological functions such as food intake and thermogenesis, as well as development of diseases such as atherosclerosis and carcinogenesis. Abnormal level of leptin and dysregulation of leptin signaling have been identified to be crucial players in pathogenesis of HCC, contributing to the malignant development and progress of obesity-related liver cancer [6–8]. Leptin signaling starts with binding to its long form receptor, and mostly activates Janus kinase (JAK) / signal transducers and activators of transcription 3 (STAT3) pathway [9]. Following nuclear translocation, STAT3 binds to DNA as a transcriptional factor, and promotes cellular proliferation and reduces apoptosis [10]. In normal cells STAT3 signal is controlled by suppressor of cytokine signaling proteins 3 (SOCS3), and down-regulation of SOCS3 is responsible for constitutive activation of STAT3 in HCC [11–13].

Epidemiological data indicate that men have 3–5 times the risk of developing HCC compared with women, suggesting that sex hormones play a role in such gender disparity in HCC development [14]. Whether estrogens play a protective or destructive role in HCC is under debate. Evidence has shown that estrogens suppress progression of fibrosis, tumor growth, and carcinogenesis in HCC [15,16]. Estrogens act on both nuclear and membrane ERs to mediate estrogenic actions. Expression of ER-α and ER-β has been reported in many types of liver cancer cells and tissues [17–19]. ER-α is usually considered as a proliferation activator in many reproductive cancer cells, including breast, ovarian, and endometrial cancers in females [20,21]. ER-β is less abundant in liver cells compared with ER-α [22]. Decreases in levels of gene expression and protein of ER-β have been found in many cancers, such as breast cancer, prostate cancer, and ovarian cancer [23–25]. The membrane-bound G protein-coupled ER (GPER) plays significant roles in many physiological and pathophysiological activities [26]. The biological importance of GPER is inconsistent among different tissues and organs. For example, GPER activation has been shown to stimulate proliferation of endometrial cancer cells [27,28], ovarian cancer cells [29], and ER-negative breast cancer cells [30]. There is also contradictory evidence that activation of GPER stimulates caspase-dependent apoptosis [31] and suppresses cancer cell proliferation via blocking tubulin polymerization and disrupting spindle formation of ovarian cancer cells [32], and inhibiting cell cycle progression in G2/M phase and thus arresting cells at G2 phase of mitosis of ovarian cancer cells [31] and prostate cancer cells [33]. The biological function and significance of different subtypes of ERs, especially ER-β and GPER, in HCC development remain largely unknown.

HepG2 cell line is the most commonly used liver cancer cell line in metabolic studies. In general, obesity-related liver cancer does not involve any viral infection. Different from many other liver cancer cell lines, such as Hep3B, Huh7 and HA22T/VGH, HepG2 cells are poor host cells for supporting replication of hepatitis B virus or hepatitis C virus [34–36], and thus is appropriate for studying the interaction of obesity hormone leptin and estrogens in liver cancer cell growth. In this study, we applied HCC cell line HepG2 cells with 17 β-estradiol (E2), the most potent physiological estrogen, and selective agonists of ERs to investigate the roles of E2 and ER subtypes on leptin-induced HCC development. We demonstrated that E2 attenuated leptin-induced HepG2 growth associated with suppressing proliferation and promoting apoptosis, interfering leptin-induced STAT3 and leptin-suppressed SOCS3, and increasing ERK and p38/MAPK signaling. Surprisingly and clinically importantly, activation of three different subtypes of ERs showed distinct results. GPER was more potent than ER-β, which was more potent than ER-α, in suppressing proliferation and inducing apoptosis. Activation of ERα or GPER reduced leptin-induced STAT3 signaling and increased ERK signaling without interfering SOCS3 or p38/MAPK signaling, while activation of ER-β diminished leptin-induced STAT3 signaling and enhanced SOCS3 and p38/MAPK signaling without affecting ERK signaling.

Interestingly, activation of ERα enhanced ERK signaling but did not promote cell proliferation, as it usually does in some types of cancer cells. Taken together, our findings provided an association among obesity hormone leptin, estrogen, and estrogen receptors in HepG2 cells.

## Materials and Methods

### Reagents

ER-α selective agonist 4,4’,4”-(4-propyl-[1H]-pyrazole-1,3,5-triyl) trisphenol (PPT), ER-β selective agonist 2,3-bis(4-hydroxy-phenyl)-propionitrile (DPN), and GPER selective agonist rel- 1- [4- (6- bromo- 1, 3- benzodioxol- 5- yl)- 3aR, 4S, 5, 9bS- tetrahydro- 3H- cyclopenta[c]quinolin- 8- yl]- ethanone (G-1) were purchased from Fisher (Waltham, MA) and Cayman Chemical (Ann Arbor, MI). E2 suitable for cell culture was purchased from Sigma-Aldrich (St. Louis, MO). All chemicals were first dissolved in DMSO and then diluted to final concentration using cell culture medium. Human leptin was purchased from National Hormone & Peptide Program (Torrance, CA).

### Cell culture and treatments

The human hepatocellular cancer-derived cell line HepG2 was obtained from American Type Culture Collection (ATCC; Manassas, VA) and maintained in phenol red-free DMEM supplemented with 10% (v/v) heat-inactivated and charcoal-stripped FBS and 1% antibiotics of 50 U/ml penicillin and 50 μg/ml streptomycin (Invitrogen, Grand Island, NY) in a 37°C cell culture incubator. The initial cell concentration was 1 × 10^5^ /ml. When cells were 70%-80% confluent, culture medium was starved in low serum (0.1% v/v FBS) for 16 h prior to experiments. Cells were treated with vehicle (1 μM dimethyl sulphoxide [DMSO]) as control, leptin (100 ng/ml, similar to the circulating leptin level of obese humans [37]), serial concentrations of E2 (from 0 nM to 1000 nM), or combination of constant leptin (100 ng/ml) and serial concentrations of E2 for 48 hours.

To examine the roles of different ERs involved in leptin signaling in HepG2 cells, cells were treated with 1 μM DMSO (no agonist), PPT, DPN, or G-1, respectively. The dose of 1 μM for selective ER agonists is commonly used in liver cancer cells [38], as well as other non-reproductive cancer cells with lower expression of ERs than reproductive cancer cells [22], such as adrenal carcinoma cells [39,40], medulloblastoma cells [41], thyroid carcinoma cells [42], and colon cancer cells [43].

### RNA interference

HepG2 cells were grown to 70% confluency in 6-well plates in phenol-red free DMEM supplemented with 5% charcoal-stripped FBS. HepG2 cells were then transfected with one of small interfering RNAs (siRNA) specific for ER-α, ER-β and GPER or negative control siRNA (Santa Cruz Biotechnology, Santa Cruz, CA), according to the manufacturer’s transfection protocol. For all the experiments, transfected HepG2 cells were harvested 48 hours after transfection.

### Cell counting and viability assay

HepG2 cell growth was evaluated after being treated for 48 hours in vitro by light microscopy HCC cell number and cell viability were measured using TC10TM automated cell counter (Bio-Rad, Hercules, CA) that counts cells within a 6–50 μm cell diameters range.

### Quantification of cell proliferation assay by bromodeoxyuridine (BrdU) incorporation

BrdU incorporation analysis was performed using an enzyme-linked immunosorbent assay (ELISA) kit (Millipore Corporation, Billerica, MA). Approximately 5 × 10^4^ HepG2 cells were seeded in a sterile 96-well tissue culture plate for 24 h. Subsequently, BrdU was added to the HepG2 cultures for 4 h. HepG2 cells were fixed and DNA denatured. The prediluted BrdU detection antibody conjugated with peroxidase at a 2000X concentrate binds to the newly BrdU incorporated cellular DNA. The resultant immune complexes were quantified by spectrophotometer microplate reader set at 450/550 nm double wavelength. Relative light units/second is proportional to amount of DNA synthesis and number of proliferating cells.

### Quantitative real-time PCR

Total RNA was isolated from cells collected 48 h after vehicle or leptin treatment using a RNeasy Plus kit (Qiagen, Foster City, CA), and was reverse transcribed using a cDNA synthesis kit (Bio-Rad). The primers synthesized by Integrated DNA Technologies (San Jose, CA) were: *Era* (accession#: NM_000125) forward 5’-GGAGGGCAGGGGTGAA-3’ and reverse 5’-GGAGGGCAGGGGTGAA-3’; *Erb* (accession#: NM_001214902) forward 5’-TTCCCAGCAATGTCACTAACTT-3’ and reverse 5’-TTGAGGTTCCGCATACAGA-3’; *Gper* (accession#: NM_001039966) forward 5′-AGTCGGATGTGAGGTTCAG-3′ and reverse 5′-TCTGTGTGAGGAGTACAAG-3′; and β-actin (accession#: NM_001101) forward 5’-AGAGCTACGAGCTGCCTGAC-3’ and reverse 5’-AGCACTGTGTTGGCGTACAG-3’. Quantitative real-time PCR was carried out using SYBR green master mixes and an iCycler (Bio-Rad). The amplified products were confirmed via gel electrophoresis and melt curve analysis. Results for each subtype of ERs were generated from triplicate experiments, calculated by a 2-ΔΔCt method, and normalized using a housekeeping gene β-actin. For each subtype of ERs, the expression level from the leptin treatment group was expressed as fold change relative to the vehicle treatment group.

### Western blotting

Protein was extracted by homogenization using lysis buffer with sodium orthovanadate, phenylmethylsulf inhibitor (Santa Cruz Biotechnology, Santa Cruz, CA), and phosphatase inhibitor cocktail (Sigma-Aldrich). Protein lysates were resolved in 4%-15% tris-glycine gels and transferred to a nitrocellulose membrane (Bio-Rad). Cleaved-caspase 3 and total caspase 3, phosphorylated and total ERK, phosphorylated and total p38/MAPK, phosphorylated and total STAT3, SOCS3, β-actin (1:1000; Cell Signaling, Danvers, MA), and ER-α, ER-β, and GPER (1:200; Santa Cruz, Dallas, TX) were detected by immunoblotting via chemiluminescence (Amersham^™^ ECL^™^ Prime, GE Healthcare). Protein band density was visualized and quantified using an Odyssey Infrared Imaging System (LI-COR, Lincoln, NE). Quantitative densitometric values of each protein of interest were normalized to β-actin or to the non-phosphorylated form of the protein.

### Statistical analysis

All data were presented as Mean ± SEM. All measurements were repeated for at least three independent experiments. Statistical analyses were performed using Prism5 GraphPad Software (La Jolla, CA). Two-way ANOVA comparing treatments with leptin and E2 or ER agonists followed by Bonferroni posttest was used to analyze cell numbers, proliferation and apoptosis, and intracellular signaling protein levels. One-way ANOVA followed by Tukey posttest was used to compare gene expression of ERs from groups treated with or without leptin. Statistically significance was set at p < 0.05. Exact probabilities and test values were omitted for simplicity and clarity of the presentation of the results.

## Results

### E2 and ER agonists opposed leptin-induced increase in cell number

**Fig 1:**
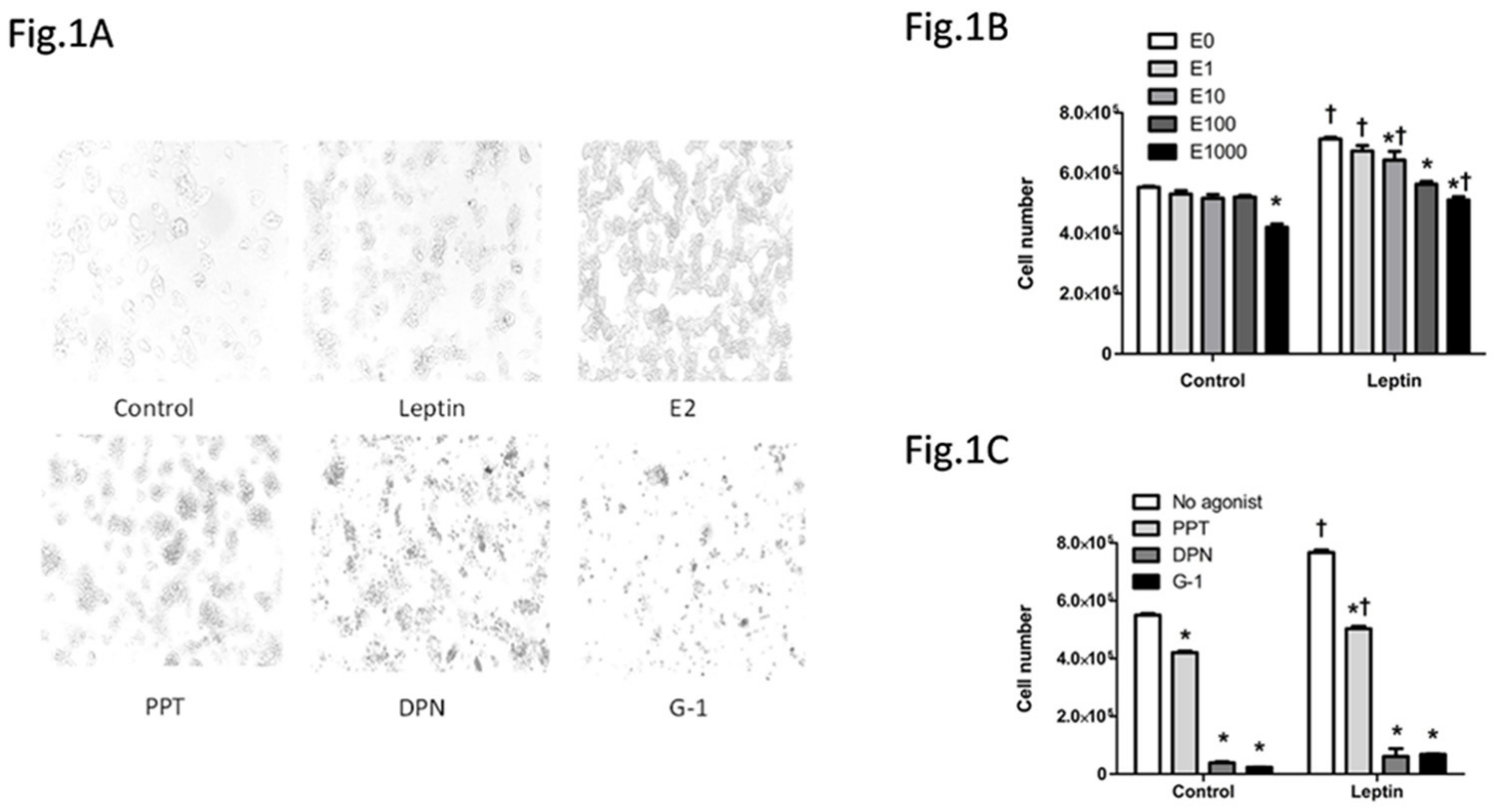
Effects of leptin, 17β-estradiol (E2) and estrogen receptor (ER) agonists on HepG2 cell number in cell culture. (A) HepG2 cells with different treatments, including vehicle DMSO (1μM) as control, leptin (100 ng/ml), E2 (1000 nM), ER-α selective agonist PPT (1 μM), ER-β selective agonist DNP (1 μM), and GPER selective agonist G-1 (1 μM), were evaluated after being treated for 48 hours using light microscopy (10 × magnification). (B, C) HepG2 cell numbers with different treatments. *: Significantly different comparing to E0 or no agonist group within the same leptin treatment (p < 0.05); †: Significantly different comparing to vehicle group within the same E2 or ER agonist treatment (p < 0.05).

Leptin increased whereas the highest dose of E2 tested (1000 nM) decreased the numbers of HepG2 cells compared with control treatment (Fig 1A and 1B). Additionally cell numbers were greater in presence of leptin comparing to vehicle, except for the 100 nM E2 groups, indicating that the increase in cell number by leptin was blocked when the cells were treated with a combination of leptin and E2 at 100 nM (Fig 1B). Further investigation of the effects of different ER agonists showed that cell numbers were decreased by PPT, DPN or G-1 compared with control (Fig 1A and 1C). Cells numbers were similar between the vehicle and leptin groups for the cultures treated with DPN or G-1, indicating that ER-β selective agonist DPN and GPER selective agonist G-1 blocked leptin-induced increase in cell number (Fig 1C).

### E2 and ER agonists decreased leptin-induced cell proliferation

**Fig 2:**
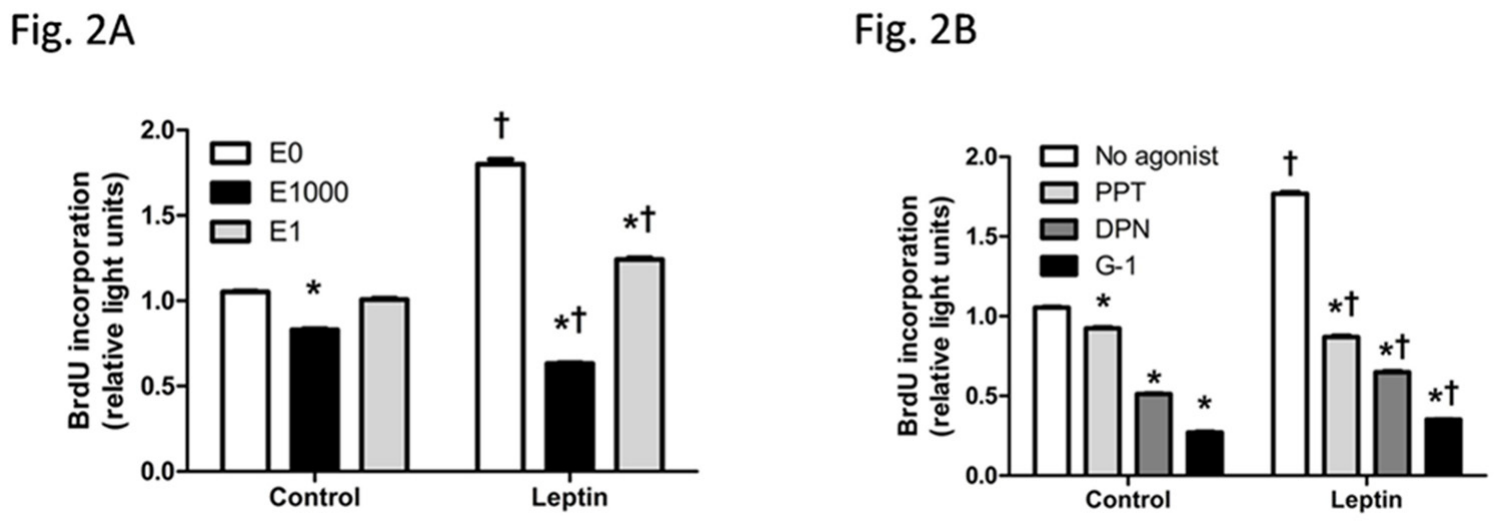
Effects of leptin, 17β-estradiol (E2), and estrogen receptor (ER) agonists on proliferation of HepG2 cells, indicated by BrdU incorporation. (A, B) BrdU incorporation assay was performed on HepG2 cells with different treatments. *: Significantly different comparing to E0 or no agonist group within the same leptin treatment (p < 0.05); †: Significantly different comparing to vehicle group within the same E2 or ER agonist treatment (p < 0.05).

Cell proliferation affects cell number and thus was assessed. E2 at 1000 nM but not 1 nM reduced cell proliferation, indicated by lower BrdU incorporation at 1000 nM E2 than control. Leptin promoted BrdU incorporation and thus HepG2 cell proliferation. Leptin-induced cell proliferation was reduced when cells were co-treated with E2 at 1000 nM or E2 at 1 nM (Fig 2A). Additionally, comparing to vehicle groups, cell proliferation was greater when cells were co-treated with leptin and 1 nM E2, but was less when cells were co-treated with leptin and 1000 nM E2 (Fig 2A), indicating that E2 at a high dose not only inhibited cell proliferation when treated alone but also opposed leptin-induced cell proliferation.

Cell proliferation was inhibited by ER-α agonist PPT, ER-β agonist DPN, or GPER agonist G-1. The increase in cell proliferation by leptin was reduced when cells were treated with leptin in combination with each of the ER agonists. Furthermore, G-1 had the least proliferative effect when either treated alone or combined with leptin (Fig 2B). Thus, treatment of each of ER agonists alone suppressed cell proliferation, and each of ER agonists was able to oppose leptin-induced cell proliferation.

### E2 and ER agonists promoted cell apoptosis

**Fig 3:**
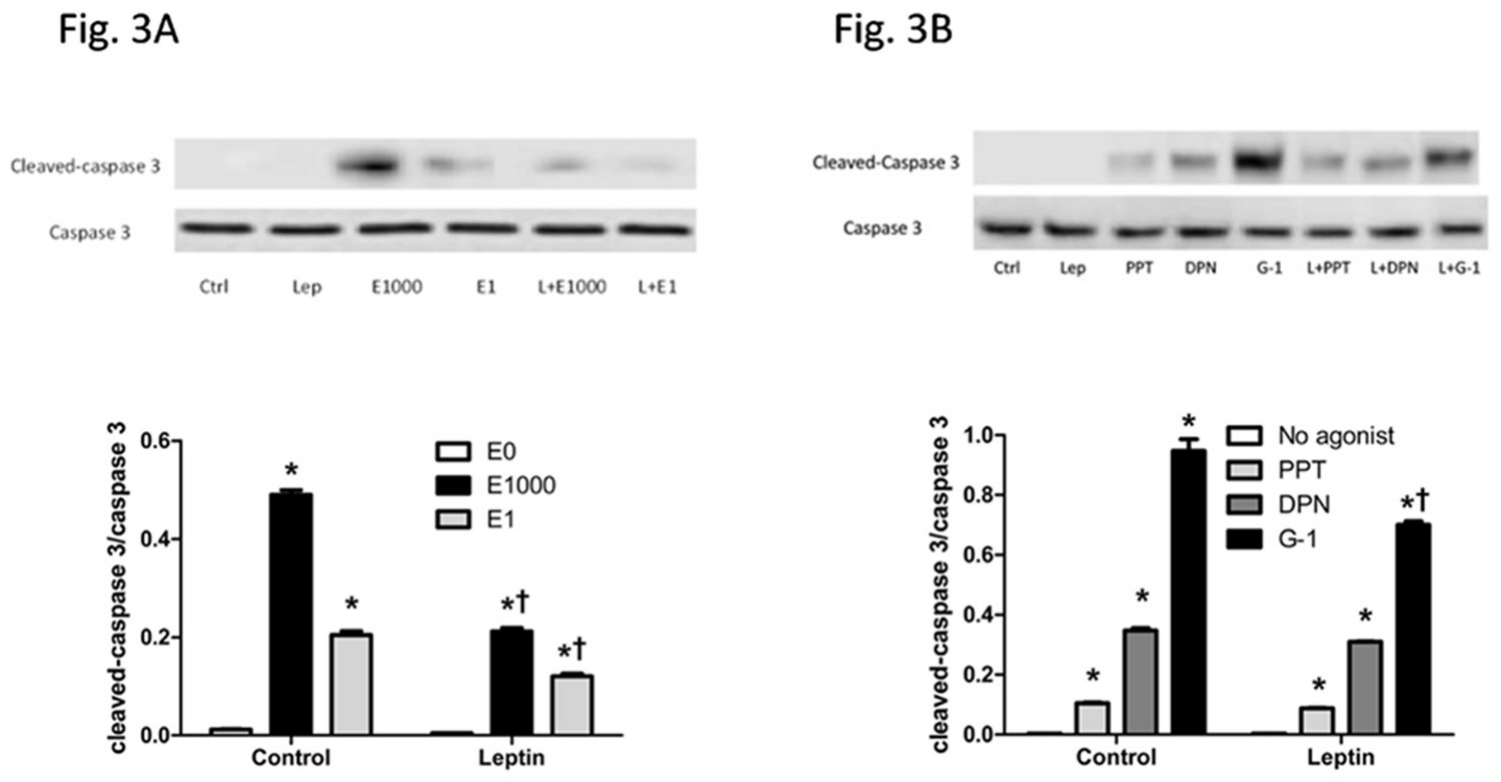
Effects of leptin, 17β-estradiol (E2), and estrogen receptor (ER) agonists on apoptosis of HepG2 cells, indicated by protein levels of cleaved-caspase 3 and total caspase 3. (A, B) Western blot of protein extracts from HepG2 cells with different treatments. *: Significantly different comparing to E0 or no agonist group within the same leptin treatment (p < 0.05); †: Significantly different comparing to vehicle group within the same E2 or ER agonist treatment (p < 0.05).

Number of cells is a net result of cell proliferation and apoptosis, thus apoptosis was assessed next. Leptin treatment alone did not affect protein level of cleaved-caspase 3, a critical executioner in apoptotic cells responsible for proteolytic cleavage of many key proteins [44]. In contrast, either with or without the presence of leptin, caspase 3 cleavage was increased following E2 treatment at 1000 nM or 1 nM, with a greater increase induced by 1000 nM E2 than 1 nM E2. Furthermore, for both dose of E2 tested here, E2-induced increase in caspase 3-dependent apoptosis was less evident with the presence of leptin than without leptin (Fig 3A).

Cleaved-caspase 3 level was increased by treatment of PPT, DPN, or G-1 alone, or by co-treatment with leptin and each of these ER agonists (Fig 3B). Additionally either with or without the presence of leptin, G-1 had greatest while PPT had least apoptotic effects (Fig 3B), indicating that GPER and ER-β play more important roles in regulating cell apoptosis than ER-α. Furthermore, G-1-induced less increase in caspase 3 cleavage with the presence of leptin than when G-1 was treated alone, whereas caspase 3 cleavage was similar between vehicle and leptin groups for PPT and DPN (Fig 3B). Thus, E2 and its receptor agonists promoted caspase 3-dependent apoptosis in HepG2 cells.

### Leptin treatment increased expression of ER-β in HepG2 cells

**Fig 4:**
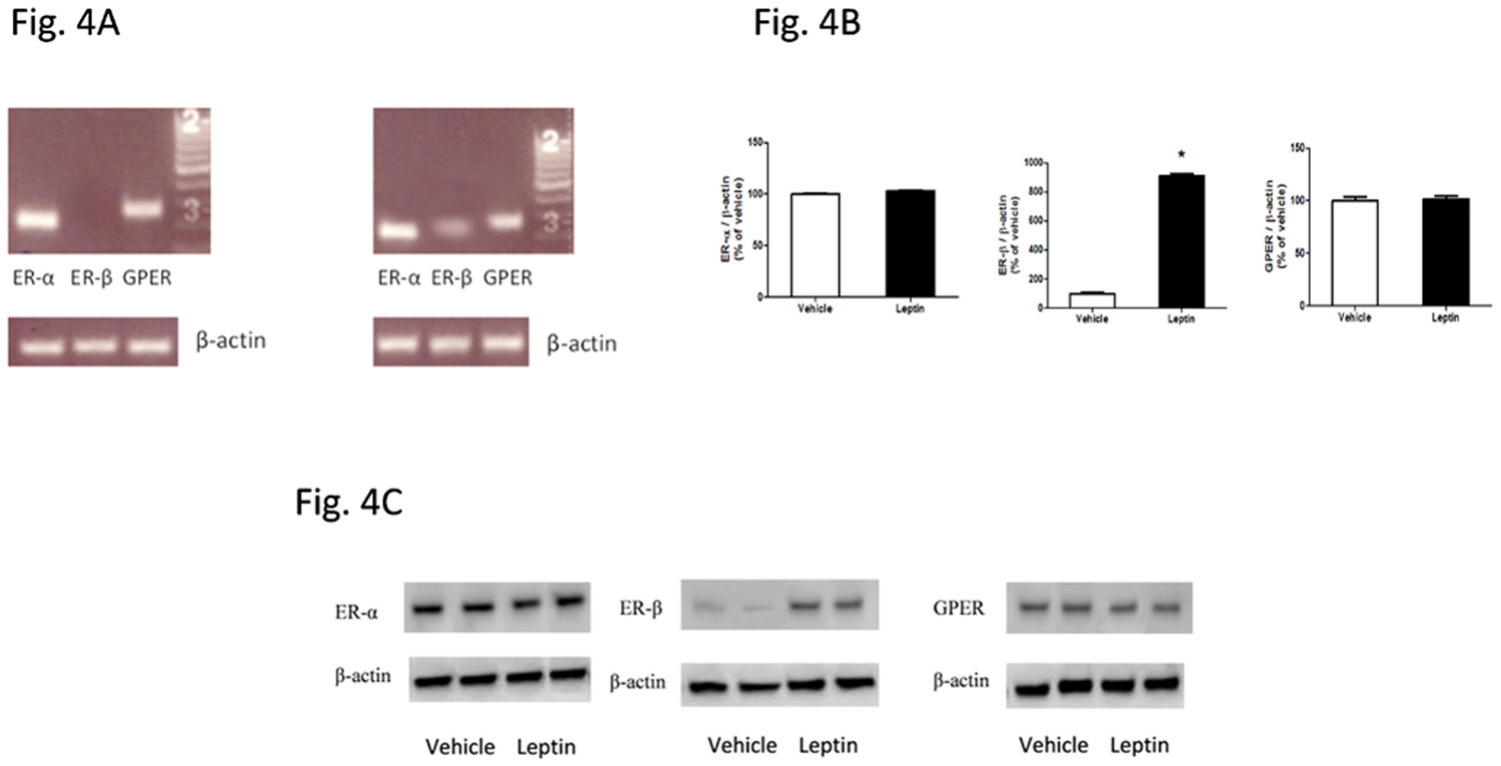
Effects of leptin treatment on estrogen receptor expression in HepG2 cells. HepG2 cells were treated without leptin or with leptin (100 ng/ml) for 48 hours. Quantitative real time-PCR and western blot were performed to measure mRNA levels and protein levels of ER-α, ER-β and GPER. (A) Quantitative real time-PCR products were verified with 1.5% agarose running gel. (B) mRNA levels of ER-α, ER-β and GPER. ER expression was normalized to β-actin and presented as fold change relative to the vehicle group. (C) Western blot images of ER-α, ER-β and GPER following treatment with vehicle or leptin. *: Significantly different ER-β mRNA levels between vehicle and leptin groups (p < 0.05).

To begin to understand the effects of E2 and its receptors in HCC development, the expressions of different subtypes of ER in HepG2 cells with or without leptin treatment were examined. Quantitative real-time PCR analysis demonstrated that the mRNA level of ER-α or GPER was similar between vehicle- and leptin-treated HepG2 cells, whereas ER-β expression in HepG2 cells was significantly higher following leptin treatment than vehicle treatment (Fig 4A and 4B). Furthermore, Western blot analysis demonstrated that protein level of ER-α or GPER was similar between vehicle- and leptin-treated HepG2 cells, whereas ER-β protein level of leptin-treated cells was significantly higher than vehicle-treated cells (Fig 4C).

### E2 and ER agonists diminished activation of leptin signaling pathway

**Fig 5:**
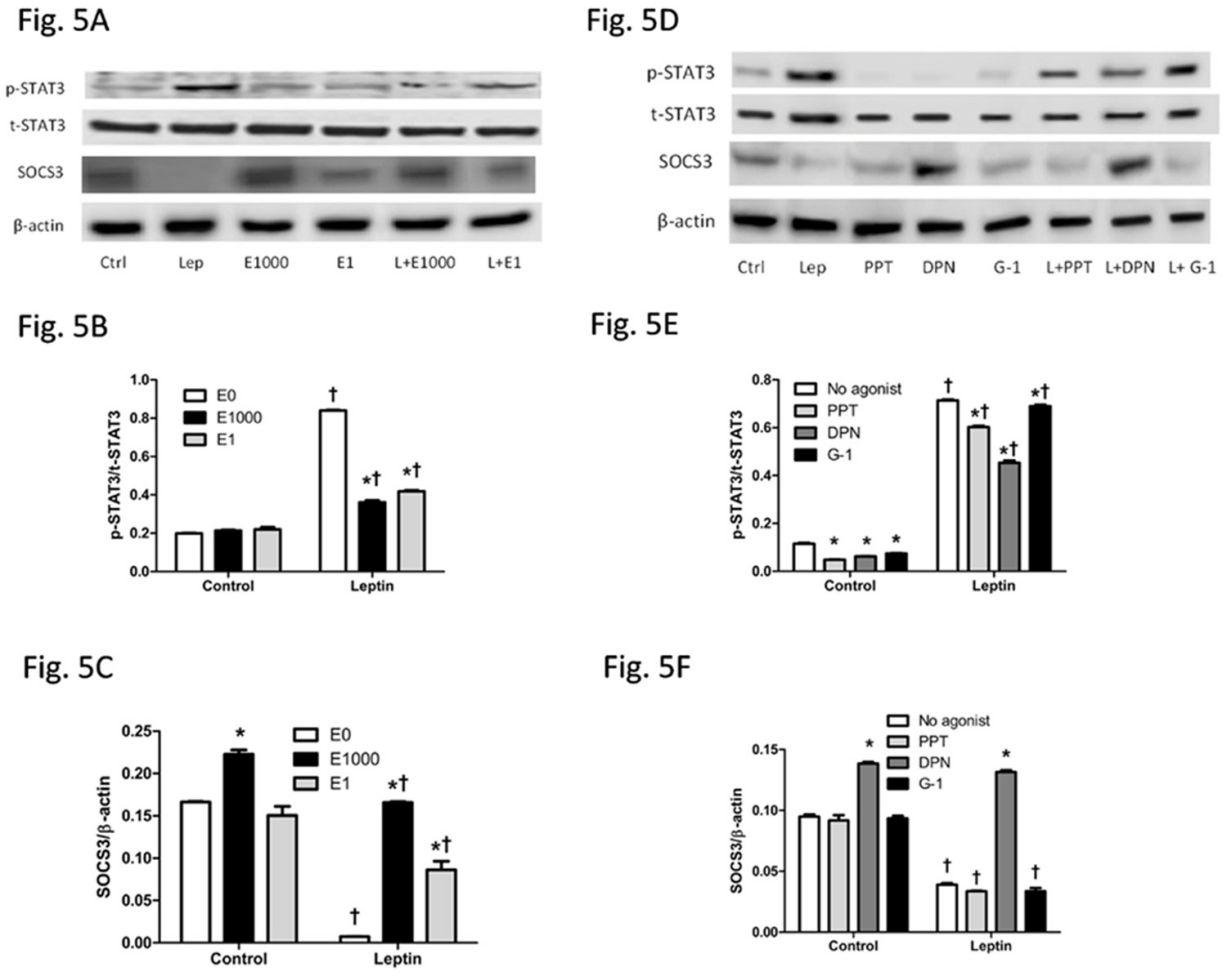
Effects of leptin, 17β-estradiol (E2) and estrogen receptor agonists on STAT3 and SOCS3 signaling in HepG2 cells. (A, D) Western blot results, (B, E) relative p-STAT3/t-STAT3 ratio, and (C, F) relative SOCS3/β-actin ratio of cells with different treatments. *: Significantly different comparing to E0 or no agonist groups within the same leptin treatment (p < 0.05); †: Significantly different comparing to vehicle groups within the same E2 or ER agonist treatment (p < 0.05).

SOCS3/STAT3, a common signaling pathway shared by many cytokines including leptin to promote cell growth and oncogenesis [45,46], was assessed. Leptin alone increased p-STAT3 and decreased SOCS3, an upstream inhibitor of p-STAT3. E2 at 1 nM alone did not affect protein levels of pSTAT3 or SOCS3 in comparison to the vehicle-treated HepG2 cells, whereas E2 at 1000 nM increased SOCS3 without changing p-STAT3. Combined treatment of leptin and E2 at 1 nM or 1000 nM significantly reduced leptin-induced p-STAT3 and increased leptin-suppressed SOCS3 (Fig 5A–5C).

Among the three different ER selective agonists, p-STAT3 level was decreased by each of ER agonists, PPT, DPN, or G-1, whereas SOCS3 signaling was increased only by DPN but not by PPT or G-1, either with or without leptin treatment (Fig 5D–5F). Although leptin-stimulated p-STAT3 was lessened by each of ER agonists PPT, DPN or G-1, p-STAT3 level of leptin-treated groups was greater than their respective vehicle treated groups (Fig 5E). Leptin-suppressed SOCS3 was blocked only by DPN but not by PPT or G-1 (Fig 5F).

### E2 and ER agonists activated ERK and p38/MAPK signaling pathways

**Fig 6:**
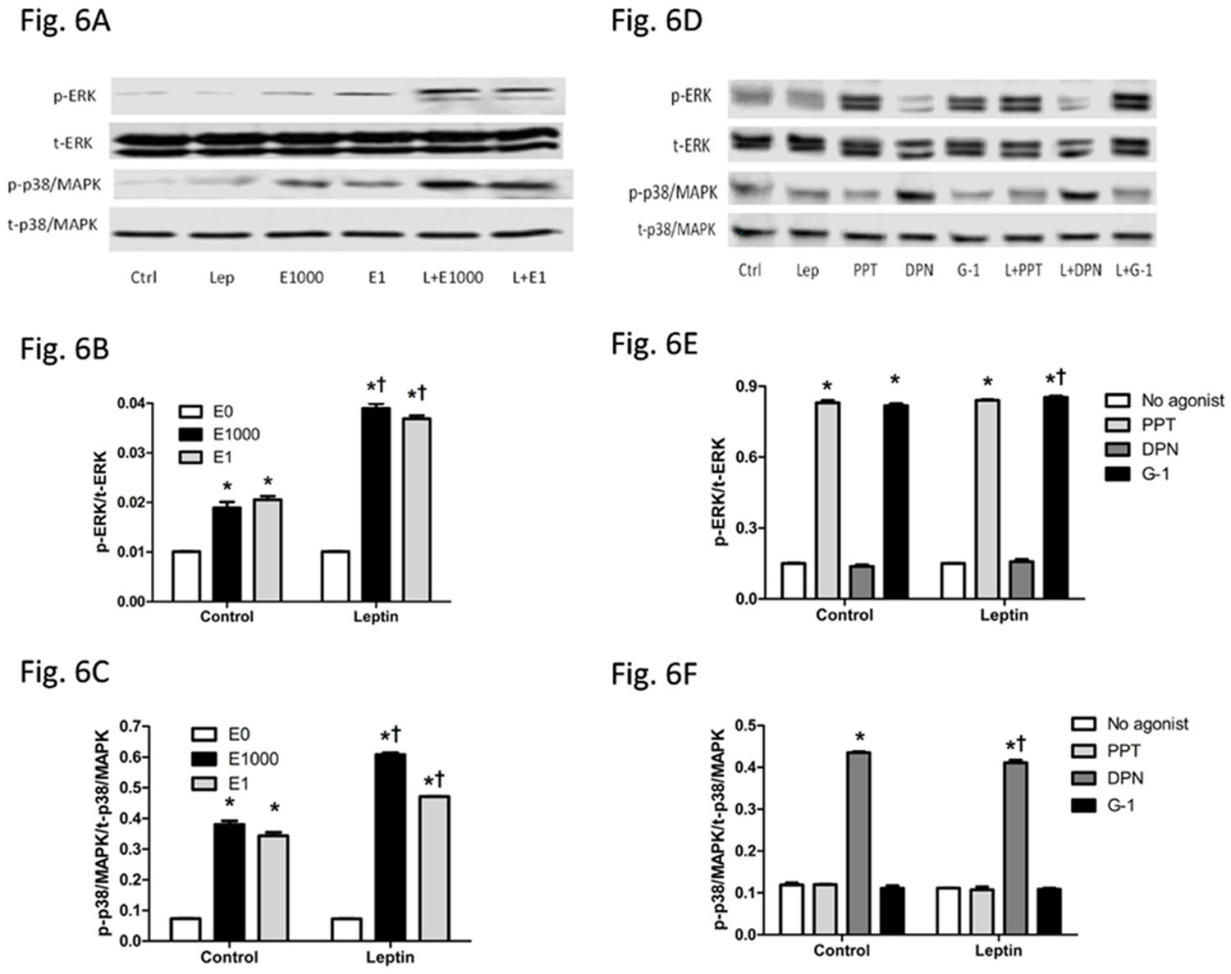
Effects of leptin, 17β-estradiol (E2) and estrogen receptor agonists on ERK and p38/MAPK signaling in HepG2 cells. (A, D) Western blot results, (B, E) relative p-ERK/t-ERK ratio, and (C, F) relative p-p38/MAPK/t-p38/MAPK ratio of cells with different treatments. *: Significantly different comparing to E0 or no agonist groups within the same leptin treatment (p < 0.05); †: Significantly different comparing to vehicle groups within the same E2 or ER agonist treatment (p < 0.05).

ERK and p38/MAPK are highly regulated in HCC development [47–49] and thus were assessed. Leptin alone did not affect pERK or p-p38/MAPK. In contrast, E2 at dose of 1 nM or 1000 nM activated both ERK and p38/MAPK. Interestingly, combination of leptin and E2 at a dose of 1 nM or 1000 nM activated ERK and p38/MAPK to a greater extent than E2 treatment alone (Fig 6A–6C). Further study using selective ER agonists revealed that PPT and G-1 activated ERK, while DPN activated p38/MAPK, either with or without leptin treatment (Fig 6D–6F). Comparing to the groups without leptin treatment, the combination treatment of G-1 with leptin significantly increased ERK activation, while the combination treatment of DPN with leptin significantly decreased p38/MAPK activation. In contrast, p-ERK level was similar between groups treated with PPT and DPN with or without leptin, and p-p38/MAPK level was similar between groups treated with PPT and G-1 with or without leptin (Fig 6D–6F).

### Loss of ER-β enhanced activation of leptin signaling pathway

**Fig 7:**
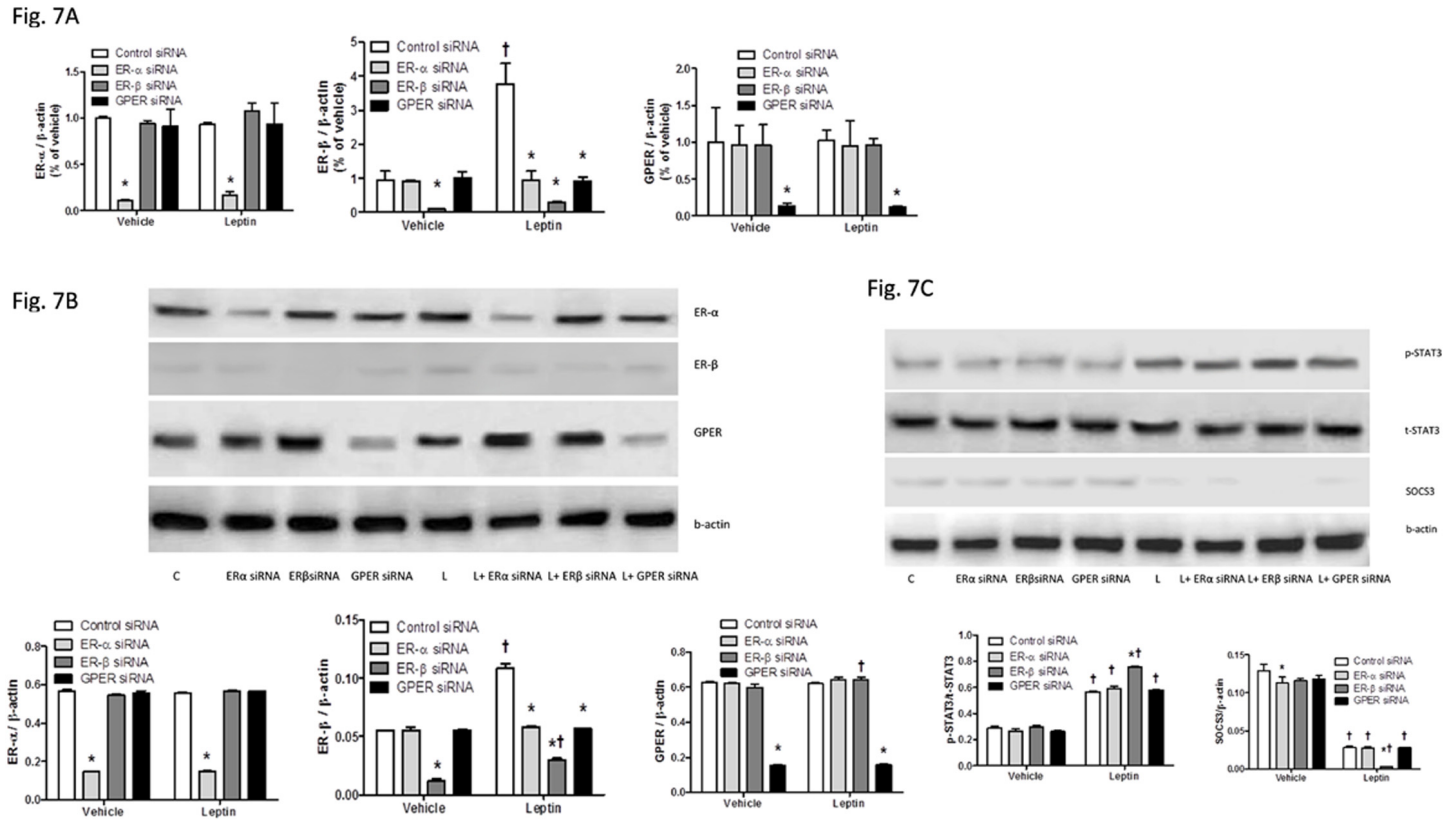
Effects of knockdown of each of specific ER subtypes using siRNAs on respective ER subtype gene expression (A), protein levels (B), and leptin signaling pathway (C). (A) Quantitative real time-PCR was performed to measure mRNA levels of ER-α, ER-β and GPER, normalized to β-actin and presented as fold change relative to the control siRNA-treated vehicle group. (B) Western blot analysis of protein levels of ER-α, ER-β and GPER, normalized to β-actin. (C) Western blot analysis of SOCS3/STAT3 signaling pathway, with relative p-STAT3/t-STAT3 ratio and relative SOCS3/β-actin ratio of cells with different treatments. *: Significantly different comparing to control siRNA groups within the same leptin treatment (p < 0.05); †: Significantly different comparing to vehicle groups within the same siRNA treatment (p < 0.05).

Since leptin signaling pathway (SOCS3/STAT3) was affected by ER agonists with or without leptin treatment (Fig 5D–5F), siRNA transfection to specific ER subtypes was used to selectively knock down each of respective ER subtype to determine role of each ER subtypes in SOCS3/STAT3 signaling pathway. Specific siRNA transfections to ER-α, ER-β, or GPER successfully inhibited both RNA transcription (Fig 7A) and protein expression (Fig 7B) of each respective ER subtype with or without leptin treatment. Additionally, ER-β siRNA, but not ER-α siRNA or GPER siRNA, enhanced STAT3 signaling and inhibited SOCS3 signaling to a greater extent than control siRNA in leptin-treated HepG2 cells (Fig 7C).

## Discussion

Leptin stimulates liver cancer development and progress [6–8], which could contribute to the high incidence of liver cancer in obese population. Estrogens and their receptors have been implicated to promote some types of cancers in women, including breast, ovarian and endometrial cancers [20,21]. Paradoxically, women have a much lower rate of liver cancer than men [14]. Although this lower incidence of HCC in women may be attributed to female sex hormone estrogens, the protective role of estrogens in leptin-induced HCC development has not been investigated yet to our knowledge. In the present study, we investigated the effects of E2 and selective agonists of ER subtypes on oncogenic action of leptin. E2 is known to induce either cell proliferation or cell apoptosis, by stimulating either oncogenes or tumor suppressor genes, depending on whether cell types are estrogen sensitive or insensitive [50–54]. The mechanisms underlying these opposite E2 effects could be partially explained by genomic vs. non-genomic estrogenic action via different ER subtypes, which then either modulates estrogen target gene transcription or rapidly activates intracellular signaling pathways, respectively [55–57]. Therefore investigating the effects of ER-α, ER-β and GPER in HepG2 cell growth to understand genomic and non-genomic estrogenic actions in liver cancer development is of great interest.

Cell number is a net outcome between cell proliferation and cell apoptosis. Leptin administration increased HepG2 cell number via enhancing cell proliferation. E2 not only increased cell apoptosis indicated by increased caspase 3 cleavage and decreased cell proliferation indicated by reduced BrdU incorporation when treated alone, but it also blocked leptin-induced increase in cell number, via stimulating apoptosis and blocking pro-proliferative effect of leptin (Figs 1–3). Liver culture cells display specific binding of E2 and with similar properties of ERs as in reproductive tissues [58] but at a much lower expression level [22]. It is noteworthy that although E2 at 1 μM has been used previously in vitro studies of liver culture cells, caution should be taken into account that E2 at a dose greater than in vivo physiological concentration would potentially impact cell growth and intracellular molecules of HepG2 cells.

Selective activation of different subtypes of ERs elicited distinct effects. The decrease in HepG2 cell number and blocking leptin-induced cell increase were the most evident by GPER selective agonist G-1 and the least evident by ER-α selective agonist PPT (Fig 1C). It is noteworthy that E2 or each of ER selective agonists suppressed proliferation and stimulated apoptosis when treated alone or co-treated with leptin in HepG2 cells (Figs 2 and 3). It has been reported that estrogen and estrogen receptor agonists can stimulate cell apoptosis in a number of cancers, including ovarian cancer [31,32]. The apoptotic effect of estrogen or its receptor agonists was not tested in hepatocellular carcinoma. In this study, we demonstrated that ER agonists alone can induce apoptosis and suppress cell proliferation in HepG2 cells. Between the two nuclear receptors ER-α and ER-β, the dominant form of ER expressed in HepG2 cells is ER-α (Fig 4). Additionally, E2 binding affinity for ER-α is higher than for ER-β [59]. Thus, E2 acts mainly via ER-α and provokes similar effects as ER-α selective agonist PPT. In contrast, DPN is an ER-β selective agonist and has a 72-fold higher binding affinity for ER-β than for ER-α [60]. DPN had far greater effects in regulating cell number than E2 (Fig 1C), suggesting that ER-β, instead of ER-α, plays major roles in suppressing cell proliferation and inducing apoptosis (Figs 2 and 3).

Despite suppressing leptin-induced cell proliferation (Fig 2A), 1 nM E2 did not significantly affect cell number in leptin-treated group (Fig 1B). Two possibilities may cause such discrepancy. First, BrdU incorporation into replicating DNA of the cells in S phase of the cell cycle was measured during a 4-hour period to indicate cell proliferation, which suggests, but does not necessarily correlate to total number of cells grown during a 48-hour period. Second, although typical mature liver cells have diameters of 20–30 μm, within the measuring range of 6–50 μm, some newly divided cells might have smaller size with diameters less than this measuring range and thus were not counted.

Among all three ER agonists tested, G-1 had the least proliferative effect (Fig 2B) and the greatest apoptotic effect (Fig 3B) when either treated alone or with leptin. These findings are consistent with previous studies showing that GPER selective agonists disrupt spindle formation and thus arrest cell cycle at prophase of mitosis in ovarian cancer [31], and induce caspase- dependent and independent programmed cell death [61,62].

We observed much lower expression of ER-β than other ER subtypes of vehicle-treated HepG2 cells (Figs 4 and 7), consistent with previous finding from other studies in the literature [63,64], and increased expression of ER-β when HepG2 cells were treated with leptin for 48 h (Fig 4), a novel finding that was demonstrated for the first time. Albeit not clear whether this increased ER-β expression is unique to leptin treatment or is related to cell proliferation, such induction of ER-β expression in HepG2 cells may potentially play protective roles in leptin-induced HCC development in the presence of E2 or ER-β agonist.

The effects of E2 and selective agonists of ER subtypes on leptin-related intracellular signaling pathways were investigated to understand potential mechanisms of estrogenic protection in leptin-induced HepG2 cell growth and thus obesity-related HCC. Leptin suppresses SOCS3 and activates STAT3 signaling, which is one of the key points involved in a number of signaling pathways to promote cell growth and oncogenesis [45,46]. E2 treatment inhibited leptin-stimulated STAT3 and increased leptin-suppressed SOCS3, both of which were mostly evident via ER-β activation (Fig 5). This was further supported by the siRNA experiment that selective knockdown of ER-β, but not ER-α or GPER, enhanced leptin-induced stimulation of STAT3 and suppression of SOCS3 (Fig 7). Some previous studies have reported activation of STAT3 up-regulates anti-apoptotic Bcl-xL family [65,66], increase G1 to S phase transition by increasing inappropriate expression of cell cycle-related proteins, including cyclin D1, cyclin-dependent kinase 4, cyclin E, cyclin A [65–67]. SOCS3 gene has been found to be silenced by promotor methylation in human lung cancer, and restoration of SOCS3 suppresses cell growth and promotes cell apoptosis [68], indicating that increase in SOCS3 signaling enhances apoptosis. Inhibition of leptin STAT3 signaling, achieved by administration of leptin antagonists [69], by leptin receptor monoclonal antibodies [70], or by upregulating SOCS3 [71] would facilitate apoptosis and impede cancer cell growth. To summarize, suppression of STAT3 and activation of SOCS3 would inhibit cancer cell growth. Consistent with the literature, E2 and ER selective agonists inhibited leptin’s effects on cell growth, which was associated with suppression of STAT3 and activation of SOCS3 signaling.

Some studies have suggested that ER-α is able to increase SOCS3 in endothelial cells of the cardiovascular system, a different type of cells from HCC cells with dominant ER-α expression but little ER-β and GPER expression [72,73]. In the current study, selective agonist of ER-β, but not ER-α or GPER, increased SOCS3 protein level when treated alone or together with leptin. The elevated ER-β expression following leptin treatment may facilitate E2 or ER-β selective agonist DPN to promote apoptosis. It is noteworthy that PPT and G-1 also enhanced apoptosis (Fig 3), but may be in an SOCS3-independent manner (Fig 5). Therefore, this study suggests that ER-β is the main ER subtype responsible for estrogen-mediated inhibition of leptin-induced changes in STAT3 signaling pathway, at least partially by enhancing SOCS3.

ERK and p38/MAPK are highly regulated in HCC development [47–49]. We found that selective agonists of ER-α and GPER activated ERK while selective agonist of ER-β activated p38/MAPK (Fig 6). There is evidence showing that ERK/MAPK pathway is regulated by multiple factors in HepG2 cells. For example, protein phosphatase 5 dephosphorylates Ser ^338^ of Raf-1, the upstream regulator of ERK, inactivates ERK [74], while reactive oxygen species inactivates ERK and activates p38/MAPK to regulate cell death [75]. ER-α and ER-β share more than 96% similarity in DNA-binding region, but only 53% similarity in ligand-binding region [76], consequently inducing different signal pathways activated by ER-α and ER-β. ER-α selective agonist PPT activated ERK, but it failed to increase HepG2 cell proliferation (Fig 3B).

In general, high p38 MAPK/ERK ratio induces dormancy and stops tumor cell growth, whereas high ERK/p38 MAPK ratio favors tumor cell growth [77,78]. Previous studies have reported that p38/MAPK significantly slows cell proliferation, induces dormancy, and induces cell apoptosis [77,78]. Particularly, p38/MAPK increases Fas/CD-95 and Bax expression and subsequently activates caspase cascade to induce apoptosis [33]. In addition, activation of p38/MAPK leads to dephosphorylation of ERK downstream molecule MEK and subsequent apoptosis [79]. Findings from this study showed that ER-β selective agonist DPN was associated with an increase in p38 MAPK signaling without changing ERK signaling. The increased p38/MAPK / ERK ratio could inhibit cancer cell growth. Furthermore, p38/MAPK can enhance SOCS3 protein by stabilization of SOCS3 mRNA level [80], which could at least partially explain the protective role of ER-β in HCC progression.

Inhibition of p38/MAPK and activation of ERK, on the other hand, promotes myoblast proliferation [81]. Activation of ERK has been shown to inhibit apoptosis by suppressing function of pro-apoptotic proteins and enhancing activity of anti-apoptotic molecules [82]. Under certain conditions however, ERK activation induces apoptosis [83]. For example, some DNA-damaging agents such as doxorubicin and antitumor compounds such as benzopyrene activate ERK and promote cell death in HepG2 cell line [84,85]. ERK activation via GPER has been reported to promote endometrial carcinoma by promoting proliferation and invasion potential [27]. A study however, demonstrates that activation of ERK via stimulation of GPER inhibits cervical cancer cell growth [86]. The role of GPER in liver cancer development is not well studied. One study reveals that activation of GPER by dehydroepiandrosterone, a predominant metabolic intermediate in biosynthesis of estrogens and androgens, increases expression of ER-α and ERK signaling in HepG2, and at meantime rapidly stimulates microRNA-21 transcription whose targets are mostly tumor suppressors [19]. Whether ERK activation by PPT or G-1 in HepG2 cells would lead to pro-apoptosis or anti-apoptosis has not been well established. Findings from the current study demonstrated that PPT and G-1 were associated with decreased cell growth and enhanced ERK phosphorylation without changing p38 activation, which indicated that ERK activation by E2 via PPT and G-1 was pro-apoptosis in HepG2 cell line.

To summarize, findings from the present study supported that estrogens attenuated leptin- induced HepG2 cell growth by facilitating apoptosis, which was associated with increase in SOCS3 and p38/MAPK signaling proteins. More importantly, we found that in HepG2 cells none of ERs was pro-proliferative, as they are in breast, endometrial and ovarian cancer cells; instead, all selective agonists of ER-α, ER-β and GPER stimulated cell apoptosis. The increased ER-β expression followed by leptin treatment is possibly due to a self-protection mechanism, although such protection from HCC development is evident with presence of E2 or agonists for ER-β. In future, the effects of ERs can be further confirmed using knock-down of respective receptors. Taken together, our data provided a better understanding of the protective role of estrogen in HCC development, and suggested an attractive target of estrogen receptor in the prevention and/or treatment of leptin-induced HCC. To conclude, this study provides better understanding of estrogenic protective role in obesity related HCC development and indicates that ER-β and GPER agonists may have implications in potential HCC treatment.
